# No Difference in Pullout Strength Between a Bio-inductive Implant and a Semitendinosus Tendon Graft in a Biomechanical Study of Medial Patellofemoral Ligament Repair Augmentation

**DOI:** 10.1016/j.asmr.2023.100827

**Published:** 2024-01-23

**Authors:** Austin Wetzler, Sean McMillan, Erik Brewer, Aakash Patel, Samuel Handy, Merrick Wetzler

**Affiliations:** a Lewis Katz School of Medicine, Temple University, Philadelphia, Pennsylvania, U.S.A.; b Virtua College of Medicine and Life Sciences, Rowan University, Strafford, New Jersey, U.S.A.; c Department of Biomedical Engineering, Rowan University, Glassboro, New Jersey, U.S.A.; d Department of Orthopedic Surgery, Philadelphia College of Osteopathic Medicine, Philadelphia, Pennsylvania, U.S.A.; e Virtua Health, Voorhees, New Jersey, U.S.A.

## Abstract

**Purpose:**

To compare the pullout strength of a bio-inductive implant (BI) used to augment a medial patellofemoral ligament (MPFL) repair with the pullout strength of semitendinosus graft in a biomechanical cadaveric model.

**Methods:**

Six matched pairs of cadavers (12 knees) were used in the biomechanical testing comparing semitendinosus tendon (Semi-T) versus a BI. The Semi-T was harvested from 1 of the matched pairs. A standard double-bundle technique using 2 sockets in the upper two-thirds of the patella 15 mm apart was performed. After docking of the graft into the patella, the patella was dissected free of soft tissues and potted into a fixture to allow mechanical pull parallel to the transverse axis of the patella. The construct was pulled to failure.

**Results:**

There was no statistically significant difference in pullout strength (*P* = .77) between the BI group (249.3 ± 36.3 N) and Semi-T group (235.0 **±** 113.6 N) double-bundle constructs. In the Semi-T group, 50% of the specimens (3 of 6 knees) failed via anchor pullout and a fourth specimen failed at the suture-anchor interface (16.7%), whereas in the BI group, 16.7% of the specimens (1 of 6 knees) failed by anchor pullout. Although the Semi-T group (49.5 ± 14.1 N/mm) showed significantly greater stiffness than the BI group (13.8 ± 0.6 N/mm, *P* < .01), pullout strength in the Semi-T group was highly variable: 50% of the specimens (3 of 6 knees) with semitendinosus constructs failed at 5 mm of displacement or less via graft or anchor pullout. Maximum load, displacement at failure, stiffness, and load at 5 mm were compared between the augmented and non-augmented control specimens using a 2-tailed non-equal variance Student *t* test. For all comparisons, *P* < .05 was considered to indicate a statistically significant difference.

**Conclusions:**

In this biomechanical study, augmentation of an MPFL reconstruction using a common double-bundle technique with a BI had the same pullout strength as a semitendinosus graft using the same technique in cadaveric knees.

**Clinical Relevance:**

MPFL repair after a patellar dislocation may be inadequate to restore the strength of the native MPFL and prevent recurrent patellar instability. Recurrent instability of the patella can result in progressive injury to the soft tissue and articular cartilage of the patella and femur. It is important to study the techniques used for MPFL repair to continually improve patient outcomes. Further testing of these additional techniques and clinical studies are needed to evaluate the implants used to augment MPFL repairs.

Dislocation and instability of the patella, especially in active adolescent individuals, are common injuries. The medial patellofemoral ligament (MPFL) is the major medial stabilizer of the patella, and it acts as a checkrein to prevent lateral dislocation. The MPFL attaches on the medial side of the patella, as well as near the medial epicondyle on the femur.^1^^,^^2^ The MPFL is usually disrupted when the patella dislocates laterally. When the MPFL is disrupted, the risk of recurrence of patellar dislocation can range from 50% to 90%.^2^ Typically, patellar dislocation occurs in younger individuals aged under 30 years, with the highest incidence occurring in those aged under 21 years.^1^^,^^2^ Recurrent instability of the patella can result in progressive injury to the soft tissue and articular cartilage of the patella and femur.^1^^,^^3^ Studies have shown that simply repairing the MPFL after a patellar dislocation may be inadequate to restore the strength of the native MPFL and prevent recurrent patellar instability.^1^^,^^4^^,^^5^ Surgical techniques have been developed to repair and augment the MPFL and prevent recurrent instability. Each method used to augment the MPFL requires securing a graft to the medial side of the patella and to the attachment of the MPFL on the femur.^6^, ^7^, ^8^, ^9^, ^10^

There are multiple techniques used to augment an MPFL repair and prevent recurrent instability.^11^, ^12^, ^13^, ^14^, ^15^, ^16^ The most common technique uses a graft or implant docked in the patella and femur. There are multiple graft choices that can be used for MPFL reconstruction, including autografts, allografts, synthetic sutures, and implants. The most common graft used is semitendinosus tendon (Semi-T) allograft. Several studies have reported the ultimate load to failure of this technique using the Semi-T.^9^^,^^16^^,^^17^ This technique and these graft choices together have been shown to be at least equal in strength to the native MPFL or as much as 4 times stronger than the native MPFL.^18^ Several other studies have shown good outcomes of MPFL reconstruction and/or augmentation using synthetic ligaments^19^^,^^20^ or high–tensile strength suture tape^11^^,^^21^, ^22^, ^23^, ^24^, ^25^ in place of the Semi-T. The goal of all these techniques is not to reconstruct the MPFL but to augment and reinforce the repaired ligament.^26^, ^27^, ^28^, ^29^, ^30^

Biological grafts and implants to augment and strengthen repairs or reconstructions have been an increased focus within orthopaedics over the past decade. However, most augmentations currently available serve 1 of 2 functions: They can either add strength to the repair or aid in biological healing. A biological implant (BioBrace; ConMed, New Haven, CT) has shown the ability to provide both strength and bio-induction of native tissue.^31^^,^^32^ This implant comes in 2 different configurations: a sheet format (23 by 30 mm) and a configuration measuring 5 mm in diameter by 250 mm in length. This implant has an open-architecture bio-inductive scaffold consisting of highly porous resorbable type I collagen and a polymer known as “poly-L-lactic acid” (PLLA)^31^^,^^32^ (Fig 1).

**Fig 1:**
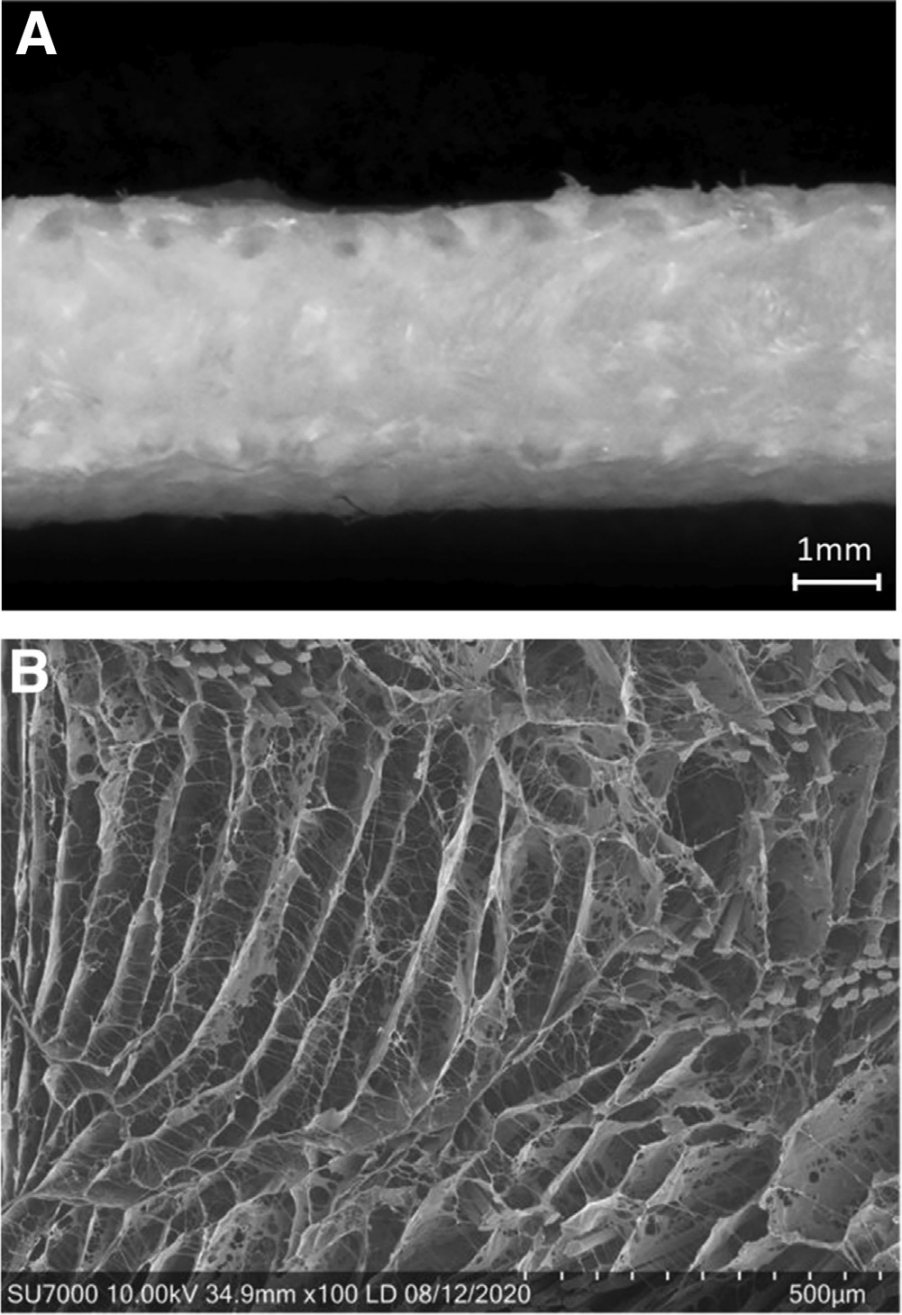
(A) Macroscopic (25×) and (B) Scanning electronic microscopic (100×) view of the bio-inductive scaffold, demonstrating 80% porous with poly (l-lactide) microfilaments reinforcement to allow for induction of host tissue, maturation, and strength.

The implant acts as a bio-inductive scaffold, and its open architecture allows for migration of the patient’s cells with induction and maturation of the host tissue. It has significant tensile strength that enables load sharing at time zero to reinforce the tissue repair during healing, and in an animal model, it has been shown to provide the strength and bio-inductive activity of native tissue.^31^^,^^32^

The bio-inductive implant (BI) (5 × 250–mm implant) can be used to augment a repair of the MPFL in the same fashion as the Semi-T allograft, as well as other synthetic grafts or suture tape. Its use would eliminate the disadvantages of Semi-T allograft most commonly used in MPFL augmentation, and it is bio-inductive whereas synthetic grafts and suture tape are not. The purpose of this study was to compare the pullout strength of a BI used to augment an MPFL repair with the pullout strength of semitendinosus (ST) graft in a biomechanical cadaveric model. The hypothesis was that the BI and Semi-T would be biomechanically similar.

## Methods

### Biomechanical Testing

**Fig 2:**
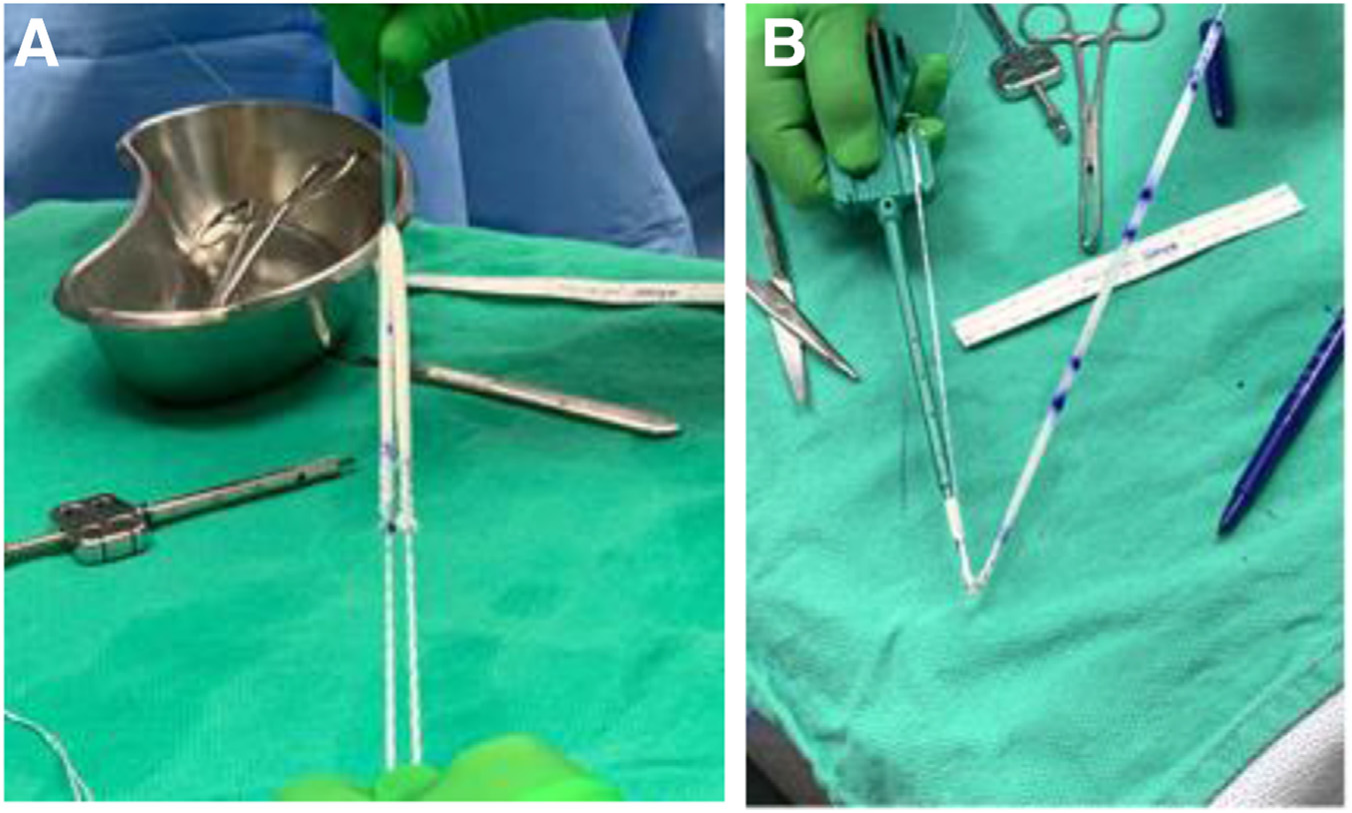
(A) The bio-inductive implant is easily whipstitched on each end and doubled over. (B) The whipstitched sutures are passed through the eyelet of the anchor and easily wrapped around and secured to the paddle of the anchor insertion handle.

**Fig 3:**
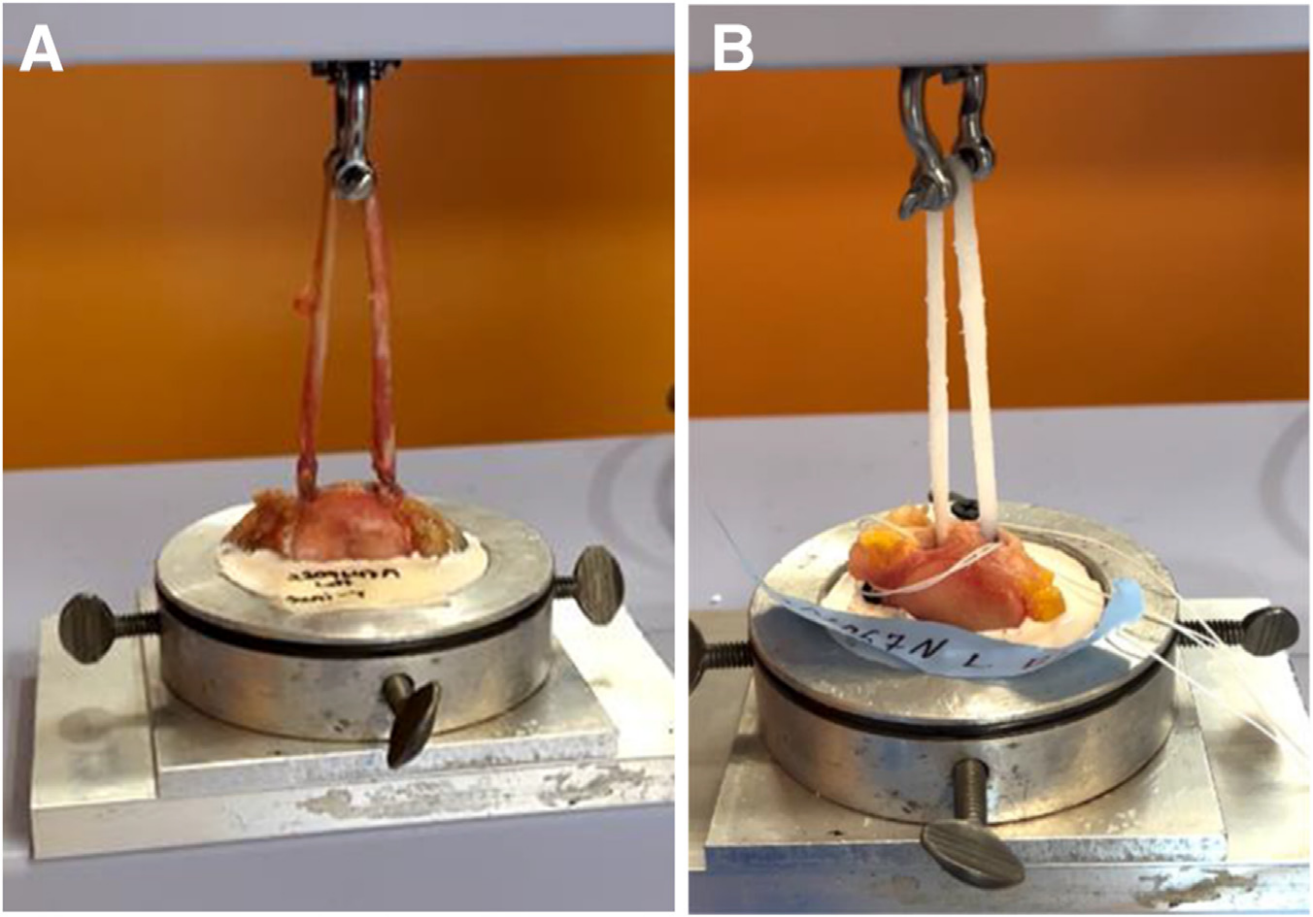
Testing setup. The looped graft is secured to the mechanical testing machine using a latch pin (Autograph AGS-X). (A) Semitendinosus. (B) Bio-inductive implant.

Six matched pairs of cadaveric knees (12 knees) without any history of surgery were obtained from MedCure (Las Vegas, NV). In this study, 6 matched pairs (12 knees) were chosen based on previous studies and power analyses performed by Gould et al.^8^ and Joyner et al.^25^ Each matched pair was randomized into the ST autograft group or BI group. For each pair of limbs, the specimens had distinct numbers associated with them. The specimen with the lower number was placed in the ST group, and that with the higher number was placed in the BI group. A Semi-T was harvested from 1 of the legs of the matched pairs and trimmed as well as possible to fit into a 4.5-mm socket. The BI was shortened to be equal in length to the harvested Semi-T grafts (220 mm). In each group, a No. 2 high–tensile strength suture was whipstitched to the ends of the ST or BI. Two sockets measuring 4.5 mm in diameter and spaced at least 1.5 cm apart were made on the medial side of the patella anterior to the articular surface. The Semi-T and BI were docked into each patellar socket using 4.75-mm anchors with the end of the graft or implant secured to an eyelet via whipstitches (Fig 2). The patella was then dissected free of soft tissues. The patella was potted into a fixture to allow mechanical pull-to-failure testing (Fig 3). The looped graft was secured to the mechanical testing machine with a latch pin (Autograph AGS-X; Shimadzu, Tokyo, Japan). The direction of pull was parallel to the transverse axis of the patella, similarly to other studies.^5^^,^^8^^,^^16^^,^^26^

Each specimen was preconditioned by a series of 10 cycles from 0 to 30 N at 1 Hz, and the length was measured again at a nominal force of 10 N. The ST and BI specimens were each pulled at 25 mm/min until failure. For each specimen, load (in newtons) versus displacement (in millimeters) was recorded at 100 Hz until failure, ultimate tensile load was recorded, and stiffness was calculated by use of a best-fit line to determine the slope of the linear portion of the load-displacement curve. The mode of failure was recorded for each specimen. Test-retest sampling was not performed because each test was a destructive test and the specimens could not be retested.

### Statistical Methods

Maximum load, displacement at failure, stiffness, and load at 5 mm were compared between the augmented and non-augmented control specimens using a 2-tailed non-equal variance Student *t* test. For all comparisons, *P* < .05 was considered to indicate a statistically significant difference.

## Results

**Fig 4:**
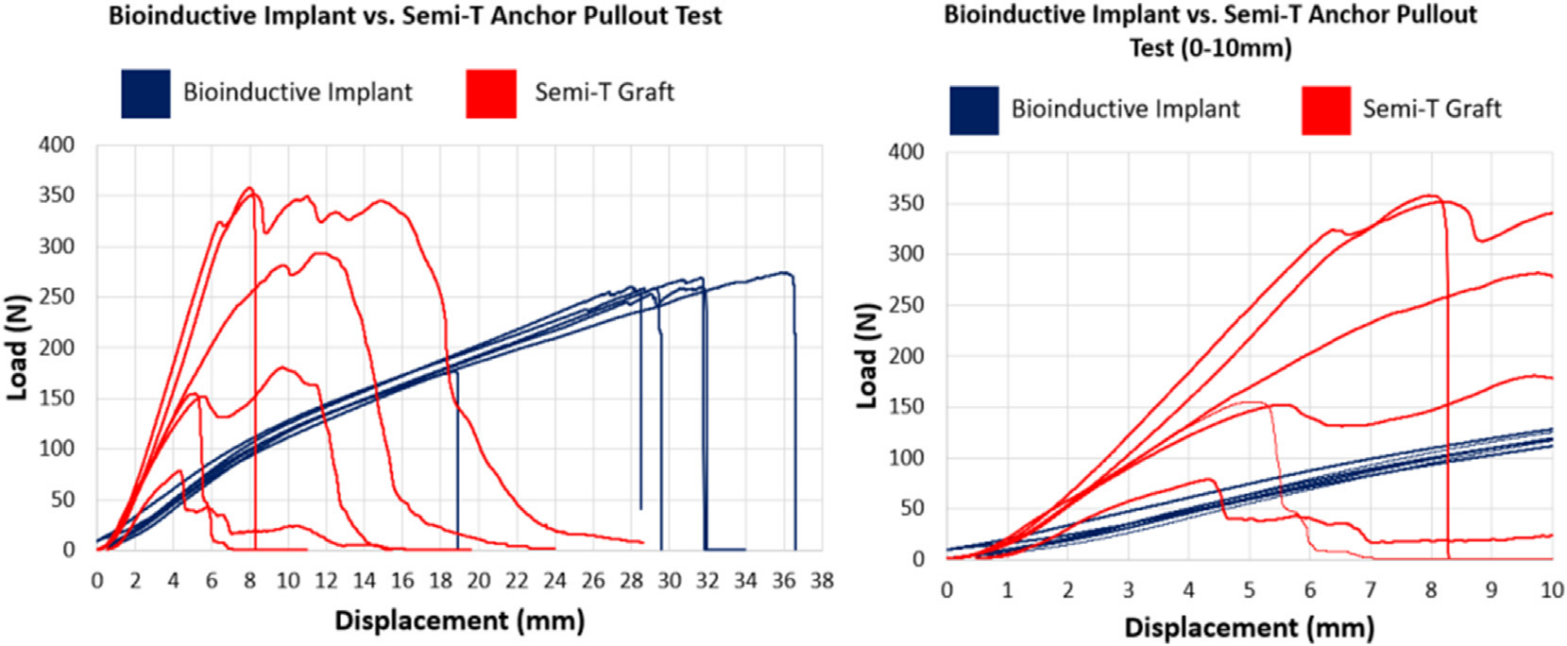
Graph of results of biomechanical testing with bio-inductive implant (blue) and semitendinosus tendon (Semi-T) (red).

There was no statistically significant difference in pullout strength (*P* = .77) between the BI group (249.3 ± 36.3 N) and ST group (235.0 **±** 113.6 N) double-bundle constructs, but the ST group had a greater than 3-fold difference in range of force (in newtons) to failure. The failure mode for both groups was either graft failure or anchor pullout. In the ST group, 4 of 6 specimens (67%) failed by anchor pullout. Although the ST group (49.5 ± 14.1 N/mm) showed significantly greater stiffness than the BI group (13.8 ± 0.6 N/mm) (*P* < .01), the pullout strength in the ST group was highly variable: 50% of the ST constructs (3 of 6 knees) failed at 5 mm of displacement or less via anchor pullout. The BI provided consistent fixation, with only 1 graft failing by anchor pullout (Tables 1 and 2, Fig 4).

**Table 1:** Comparison of Results of Biomechanical Testing of Bio-inductive Implant and Semitendinosus Graft

	Bio-inductive Implant	Semitendinosus Graft
Displacement at failure	31.5 ± 4.8	10.0 ± 4.5
Failure load, N	249.3 ± 36.3	235.0 ± 113.6
Stiffness at 5 mm of displacement, N/mm	13.8 ± 0.6	49.5 ± 14.1
Load at 5 mm of displacement, N/mm	63.0 ± 6.6	162.1 ± 71.5

NOTE. Data are presented as mean ± standard deviation.

**Table 2:** Statistical Significance of Measured Parameters of Biomechanical Testing

Parameter	*P* Value	Conclusion
Displacement at failure	<.0001	The semitendinosus graft fails at a lower amount of displacement.
Maximum load	.77	There is no significant difference in maximum load.
Stiffness at 5 mm of displacement	.0002	The semitendinosus graft is stiffer than the bio-inductive implant.
Load at 5 mm of displacement	.007	The semitendinosus graft has a higher load than the bio-inductive implant at 5 mm.

## Discussion

The most important finding of this study was that a BI had the same pullout strength as an ST graft using the same technique in cadaveric knees. The results of biomechanical testing showed that the mode of failure for both groups was either graft failure or anchor pullout. The ST group showed significantly greater stiffness than the BI group. However, the ST group’s pullout strength was highly variable, and 50% of the constructs (3 of 6 specimens) failed at 5 mm of displacement or less by anchor pullout, which is premature compared with the native MPFL.^5^^,^^33^

**Fig 5:**
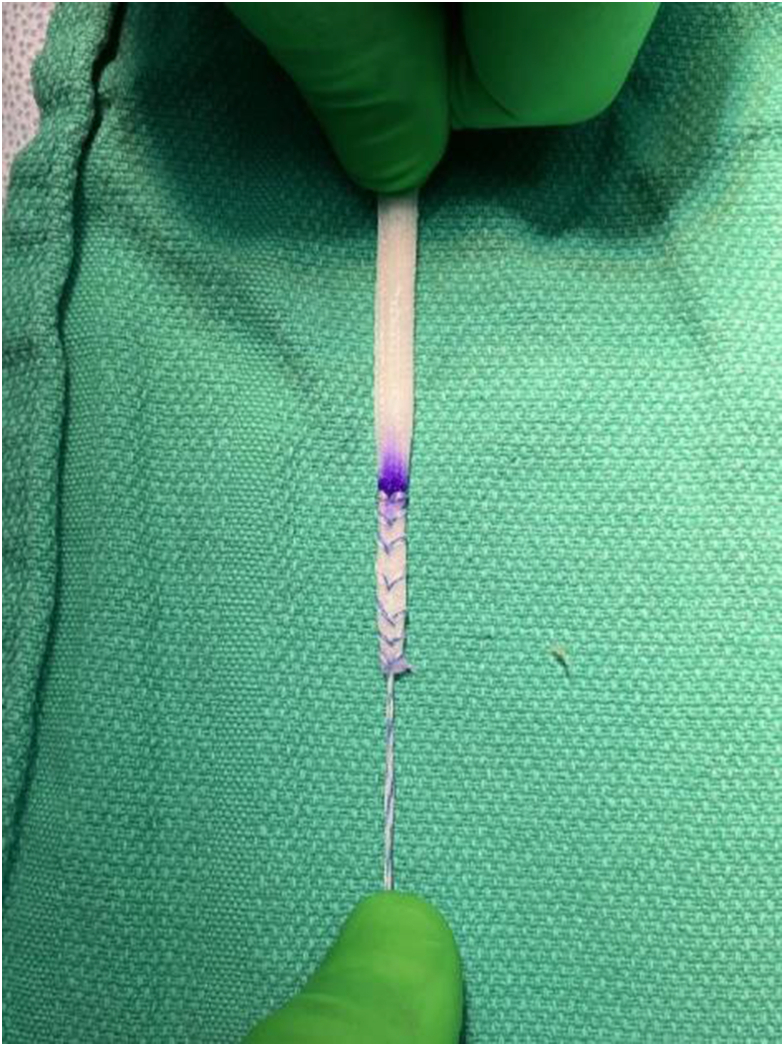
Close-up view showing whipstitched end of bio-inductive implant.

Studies have shown that simply repairing the injured MPFL yields a high recurrent dislocation rate.^2^^,^^4^ Techniques have been developed to augment MPFL repairs with various autografts, allografts,^7^^,^^10^^,^^11^^,^^13^^,^^15^, ^16^, ^17^, ^18^^,^^26^^,^^27^^,^^29^ and synthetic materials.^11^^,^^19^, ^20^, ^21^, ^22^, ^23^, ^24^, ^25^ Many different techniques and fixation methods have been biomechanically tested, and the use of an interference screw for patellar fixation appears to be the strongest.^2^^,^^6^^,^^16^, ^17^, ^18^^,^^34^ The most popular graft choice is the Semi-T, and testing has shown this graft to be the strongest or stiffest for MPFL augmentation or reconstruction^5^^,^^8^^,^^16^^,^^26^; however, the use of Semi-T graft is not without some inherent problems. Studies have shown wide variation in the lengths and cross-sectional areas of the Semi-T.^35^, ^36^, ^37^ Unlike in anterior cruciate ligament reconstruction, where the graft is doubled over and the whipstitched ends are usually not incorporated into the reconstruction, in augmentation or reconstruction of the MPFL, each end of the ST needs to be securely whipstitched and docked into 4.5-mm tunnels in the patella. During this study, difficulty was found regarding the Semi-T harvested from the matched-pair cadavers. In 3 of the specimens, the construct failed at 5 mm of displacement or less. The ends of the Semi-T had to be trimmed, there was slippage of the whipstitches through each end of the Semi-T, and there was some difficulty in docking the ends of the Semi-T in the patella. These situations may have weakened the fixation of the graft and/or anchor in the socket, resulting in premature anchor pullout and the wide variation in failure strength of the Semi-T seen in this study. The advantage of the BI is that no modification is required except shortening the graft to the appropriate length, and the whipstitch yields excellent fixation of the ends of the implant. With pretension, the tip of the implant the end becomes slightly tapered allowing easy and reproducible docking of the ends into the patellar sockets (Fig 5).

Mountney et al.^5^ have shown that the native MPFL strength is 208 N at displacement of 26 mm at failure. The results of our study showed that the BI was stronger and had nearly the same stiffness (249.3 ± 36.3 N at 31.5 ± 4.8 mm of displacement) as in the study of Mountney et al., whereas the ST (235.0 **±** 113.6 N at 10.0 ± 4.5 mm) was stronger and stiffer than the native MPFL. It is believed that we can compare our results with those of Mountney et al. because the biomechanical testing in each study was performed with a pull to failure parallel to the transverse axis of the patella^5^ (Fig 4).

Carter et al.^30^ and Walsh et al.^31^ showed in a rotator cuff animal model that the aforementioned BI allows for rapid cellular infiltration and growth of regularly oriented connective tissue, with tissue thickness increased by 180% after 6 weeks.^32^ At 12 weeks, the repair was as strong as the native tendon. The BI used in the previously mentioned studies was a flat sheet. The 5-mm-diameter BI used in our study has the same microscopic structure as the sheet. The BI has several advantages over the Semi-T. The BI is consistent in size and shape and can be reliably docked into a 4.5-mm socket without significant manipulation. Semi-T autograft or allograft almost always has to be trimmed, and it can be difficult to secure it into the 4.5-mm sockets in the patella. The allograft needs to be stored in a special freezer, whereas the BI can be used off the shelf and is stored at room temperature with no preparation (i.e., thawing or significant hydration) required. Additionally, studies have shown that allograft tissue can elicit an immune response and there could be delayed incorporation and healing of the native graft.^36^^,^^37^ Finally, reproducible and secure fixation of the BI was more consistently obtained, with biomechanical testing showing that the strength of the ST-anchor-socket interface is less consistent.

### Limitations

Limitations of this study include using matched pairs of cadaveric specimens. The bone density of these specimens was less than the bone density in adolescents and young adults who are usually the patients who experience recurrent patellar dislocation. By using matched pairs, the goal was to reduce the influence of the decreased bone density and allow us to make a comparison between the groups. The biomechanical test was not anatomic, but the pull-to-failure test was performed parallel to the transverse axis of the patella, similarly to other studies previously published.^5^^,^^8^^,^^16^^,^^26^ Only 1 technique was used, and additional biomechanical studies using other common techniques would be valuable.

## Conclusions

In this biomechanical study, augmentation of an MPFL reconstruction using a common double-bundle technique with a BI had the same pullout strength as an ST graft using the same technique in cadaveric knees.

## Disclosure

The authors report the following potential conflicts of interest or sources of funding: S.M. receives personal fees from Biorez, 10.13039/100019400ConMed, Mitek, BD, Trice Medical, and Micah, outside the submitted work; owns stock in Biorez, Trice Medical, Avalon AI, and Kaliber Technologies, outside the submitted work; and is on the board of directors of American Osteopathic Academy of Orthopedics and New Jersey State Orthopedic Society. M.W. receives grant support for cadaveric specimens from Biorez and Philadelphia College of Osteopathic Medicine; is on the editorial board of *Journal of Arthroscopy*; is a member of AANA Developmental and Membership Committee; and is AANA representative to 10.13039/100009885American Academy of Orthopaedic Surgeons Board of Specialty Societies. All other authors declare that they have no known competing financial interests or personal relationships that could have appeared to influence the work reported in this work. Full ICMJE author disclosure forms are available for this article online, as supplementary material.

## Supplementary Data

ICMJE author disclosure forms

## References

[1] Gurusamy P., Pedowitz J.M., Carroll A.N. (2021). Medial patellofemoral ligament reconstruction for adolescents with acute first-time patellar dislocation with an associated loose body. Am J Sports Med.

[2] Kyung H.S., Kim H.J. (2015). Medial patellofemoral ligament reconstruction: A comprehensive review. Knee Surg Relat Res.

[3] Hayat Z., El Bitar Y., Case J.L. (2022). *StatPearls. Treasure Island (FL): StatPearls, 2022*. Treasure Island (FL).

[4] Ambra L.F., Franciozi C.E., Phan A., Faloppa F., Gomoll A.H. (2021). Isolated MPTL reconstruction fails to restore lateral patellar stability when compared to MPFL reconstruction. Knee Surg Sports Traumatol Arthrosc.

[5] Mountney J., Senavongse W., Amis A.A., Thomas N.P. (2005). Tensile strength of the medial patellofemoral ligament before and after repair or reconstruction. J Bone Joint Surg Br.

[6] Buckens C.F., Saris D.B. (2010). Reconstruction of the medial patellofemoral ligament for treatment of patellofemoral instability: A systematic review. Am J Sports Med.

[7] Dornacher D., Lippacher S., Nelitz M. (2018). Impact of five different medial patellofemoral ligament-reconstruction strategies and three different graft pre-tensioning states on the mean patellofemoral contact pressure: A biomechanical study on human cadaver knees. J Exp Orthop.

[8] Gould H.P., Delaney N.R., Parks B.G., Melvani R.T., Hinton R.Y. (2021). Interference screw versus suture anchors for femoral fixation in medial patellofemoral ligament reconstruction: A biomechanical study. Orthop J Sports Med.

[9] He W., Yang Y.M., Liu M., Wang A.Y., Liu Y.J. (2013). Reconstruction of the medial patellofemoral ligament using hamstring tendon graft with different methods: A biomechanical study. Chin Med Sci J.

[10] Nomura E., Inoue M., Kurimura M. (2003). Chondral and osteochondral injuries associated with acute patellar dislocation. Arthroscopy.

[11] Schöttle P.B., Hensler D., Imhoff A.B. (2010). Anatomical double-bundle MPFL reconstruction with an aperture fixation. Knee Surg Sports Traumatol Arthrosc.

[12] Saper M.G., Meijer K., Winnier S., Popovich J., Andrews J.R., Roth C. (2017). Biomechanical evaluation of classic solid and all-soft suture anchors for medial patellofemoral ligament reconstruction. Am J Sports Med.

[13] Wang Q., Huang W., Cai D., Huang H. (2017). Biomechanical comparison of single- and double-bundle medial patellofemoral ligament reconstruction. J Orthop Surg Res.

[14] Zhao X., Zhang H. (2021). Biomechanical comparison of 2 patellar fixation techniques in medial patellofemoral ligament reconstruction: Transosseous sutures vs suture anchors. Orthop J Sports Med.

[15] Raoulis V.A., Zibis A., Chiotelli M.D. (2021). Biomechanical evaluation of three patellar fixation techniques for MPFL reconstruction: Load to failure did not differ but interference screw stabilization was stiffer than suture anchor and suture-knot fixation. Knee Surg Sports Traumatol Arthrosc.

[16] Russ S.D., Tompkins M., Nuckley D., Macalena J. (2015). Biomechanical comparison of patellar fixation techniques in medial patellofemoral ligament reconstruction. Am J Sports Med.

[17] Dobke L.S., Bonadiman J.A., Lopes O.V., Saggin P.R., Israel C.L., Spinelli L.F. (2020). Biomechanical study of different femoral fixation devices in the reconstruction of the medial patellofemoral ligament in porcine knees. Rev Bras Ortop (Sao Paulo).

[18] Berruto M., Ferrua P., Tradati D., Uboldi F., Usellini E., Marelli B.M. (2017). Suture anchors fixation in MPFL reconstruction using a bioactive synthetic ligament. Joints.

[19] Tucker A., McMahon S., McArdle B., Rutherford B., Acton D. (2018). Synthetic versus autologous reconstruction (Syn-VAR) of the medial patellofemoral ligament: A study protocol for a randomised controlled trial. Trials.

[20] Ishibashi Y., Kimura Y., Sasaki E., Sasaki S., Yamamoto Y., Tsuda E. (2020). Medial patellofemoral ligament reconstruction using FiberTape and knotless SwiveLock anchors. Arthrosc Tech.

[21] Mehl J., Otto A., Comer B. (2020). Repair of the medial patellofemoral ligament with suture tape augmentation leads to similar primary contact pressures and joint kinematics like reconstruction with a tendon graft: A biomechanical comparison. Knee Surg Sports Traumatol Arthrosc.

[22] Migliorini F., Eschweiler J., Spiezia F., Knobe M., Hildebrand F., Maffulli N. (2022). Synthetic graft for medial patellofemoral ligament reconstruction: A systematic review. J Orthop Traumatol.

[23] Tsushima T., Tsukada H., Sasaki S. (2019). Biomechanical analysis of medial patellofemoral ligament reconstruction: FiberTape with knotless anchors versus a semitendinosus tendon autograft with soft anchors. J Orthop Sci.

[24] Xu J-c, Zhang B-x, Jia Y-f (2021). Medial patellofemoral ligament reconstruction using suture tape for patellofemoral joint instability. Orthop Surg.

[25] Joyner P.W., Bruce J., Roth T.S. (2017). Biomechanical tensile strength analysis for medial patellofemoral ligament reconstruction. Knee.

[26] Lenschow S., Schliemann B., Gestring J., Herbort M., Schulze M., Kösters C. (2013). Medial patellofemoral ligament reconstruction: Fixation strength of 5 different techniques for graft fixation at the patella. Arthroscopy.

[27] Mehta V., Mandala C., Akhter A. (2017). Cyclic testing of 3 medial patellofemoral ligament reconstruction techniques. Orthop J Sports Med.

[28] Migliorini F., Baroncini A., Eschweiler J., Tingart M., Maffulli N. (2022). Interference screws vs. suture anchors for isolated medial patellofemoral ligament femoral fixation: A systematic review. J Sport Health Sci.

[29] Migliorini F., Trivellas A., Eschweiler J., Knobe M., Tingart M., Maffulli N. (2022). Comparable outcome for autografts and allografts in primary medial patellofemoral ligament reconstruction for patellofemoral instability: Systematic review and meta-analysis. Knee Surg Sports Traumatol Arthrosc.

[30] Carter A.J., Lovric V., Morberg P. (2021). Characterization of a novel bio-inductive biocomposite scaffold for tendon and ligament healing. *Presented at the Orthopaedic Research Society (ORS) 2021 Annual Meeting; February 12-16, 2021, Virtual*.

[31] Walsh W.R., Carter A.J., Lovric V. (2021). Tissue-engineered augmentation of a rotator cuff tendon using a novel bio-inductive biocomposite scaffold: A preliminary study in sheep. *Presented at the Orthopaedic Research Society (ORS) 2021 Annual Meeting; February 12-16, 2021, Virtual*.

[32] LaPrade M.D., Kallenbach S.L., Aman Z.S. (2018). Biomechanical evaluation of the medial stabilizers of the patella. Am J Sports Med.

[33] Sequeira S.B., Imbergamo C., Gould H.P. (2022). Interference screws are biomechanically superior to suture anchors for medial patellofemoral ligament reconstruction: A systematic review and meta-analysis. Arthrosc Sports Med Rehabil.

[34] Borchers J.R., Pedroza A., Kaeding C. (2009). Activity level and graft type as risk factors for anterior cruciate ligament graft failure: A case-control study. Am J Sports Med.

[35] Buerba R.A., Boden S.A., Lesniak B. (2021). Graft selection in contemporary anterior cruciate ligament reconstruction. J Am Acad Orthop Surg Glob Res Rev.

[36] Duchman K.R., Lynch T.S., Spindler K.P. (2017). Graft selection in anterior cruciate ligament surgery: Who gets what and why?. Clin Sports Med.

[37] Pichler W., Tesch N.P., Schwantzer G. (2008). Differences in length and cross-section of semitendinosus and gracilis tendons and their effect on anterior cruciate ligament reconstruction. J Bone Joint Surg Br.

